# Reducing stigma impacting children and adolescents in low- and middle-income countries: The development of a common multi-component stigma reduction intervention

**DOI:** 10.1371/journal.pone.0292064

**Published:** 2023-10-31

**Authors:** Kim Hartog, Ruth M. H. Peters, Racheal Kisakye Tukahiirwa, Mark J. D. Jordans

**Affiliations:** 1 Amsterdam Institute for Social Science Research (AISSR), University of Amsterdam, Amsterdam, The Netherlands; 2 Research and Development Department, War Child, Amsterdam, The Netherlands; 3 Faculty of Science, Athena Institute, Vrije Universiteit Amsterdam, Amsterdam, The Netherlands; 4 Mental Health and Psychosocial Support Department, TPO Uganda, Kampala, Uganda; Caleb University, NIGERIA

## Abstract

**Introduction:**

Stigmatisation impedes health and quality of life. Evidence regarding stigma reduction interventions is, albeit growing, limited. There is a gap in the availability and evidence of interventions for reducing stigma among children and adolescents, especially in low- and middle-income countries. This paper describes the process that led to a stigma reduction intervention impacting children and adolescents in low- and middle-income countries, following previously conducted formative research.

**Methods:**

In this study, we conducted (i) online stakeholder consultations (FGD) (n = 43), including a survey assessing intervention acceptability, appropriateness, feasibility and scalability (n = 16); and (ii) preliminary field-testing of intervention content online and in a refugee settlement in Uganda.

**Findings:**

Stakeholder consultation showed the initial version of STRETCH (Stigma Reduction to Trigger Change for Children), albeit positively received, required adaptations. We made adjustments to i) take into account implementation duration, intervention flexibility and intersectionality; (ii) strengthen the involvement of individuals, including adolescents/youth, with lived stigma experience; (iii) target people close to individuals with lived stigma experience; and (iv) address feasibility and sustainability concerns. Preliminary field-testing simplified STRETCH while adding a community outreach component and revisiting the intervention setup, to ensure STRETCH can also be applied from a modular perspective.

**Conclusion:**

We conducted a process to develop a child-focused multi-component stigma reduction intervention, with intended applicability across stigmas and settings. This paper provides an overview of the intervention development process, generating intervention-specific learnings with generic value. STRETCH aims to reduce stigmatisation at the implementing organisation, create community-wide reflection and stigma reduction demand, and reduce stigmatisation among various target groups.

## Introduction

The concept of stigmatisation developed from an individualistic undesirable and shameful trait [1] into a multi-dimensional societal process of labelling differences, attaching negative attributes, separation between ‘us’ and ‘them’, status loss, and discrimination [2]. A global phenomenon, stigmatisation occurs within a context of power [3] and is deeply rooted in socially constructed norms of what is deemed good or bad, moral or immoral in that specific setting [4]. Examples of identities facing stigmatisation include one’s ability [5], gender orientation and sexual identity [6], refugee status [7] or mental health condition [8]. Age can be a determining, intersecting factor in how some stigmatised identities are perceived; one study concluded that children with depression were perceived as more dangerous than adults [9] while other research showed that children with HIV/AIDS were perceived as more innocent than adults [10].

Detrimental consequences can include increased levels of depressive symptoms [11, 12] such as social withdrawal [13], decreased help-seeking behaviour [14, 15] and adherence to treatment [16], social rejection [17] and impaired social and academic functioning [18]. Internalised or self-stigmatisation can lead to more secrecy and avoidance [19], while experiencing stigmatisation during childhood may lead to a cumulative burden of distress [20], potentially altering the activation of the stress response system during adulthood [21]. Stigma is an important social determinant of health and health inequity [22] and people with lived stigma experience (PWLE) have described stigmatisation as worse than the disease itself [23–26]. The burden of stigma can exceed the burden of disease [27], impact child survival and health outcomes [28], and trigger suicidal ideation [29].

While the number of intervention studies has increased in recent years [30, 31], evidence-based stigma reduction interventions remain scarce [25, 32–34]. Stigma reduction regarding children and adolescents in Low- and Middle-Income Countries (LMIC) seems to be specifically under-researched [35]. This is disconcerting as 90% of the global child population resides in LMIC [36, 37], and interventions originating in High-Income Countries (HIC) may not fit the local drivers of stigmatisation, such as perspectives on recovery [38, 39] nor its available resources, for example, the constraint (health) infrastructure [38].

### Towards a child-focused stigma reduction intervention

**Fig 1:**
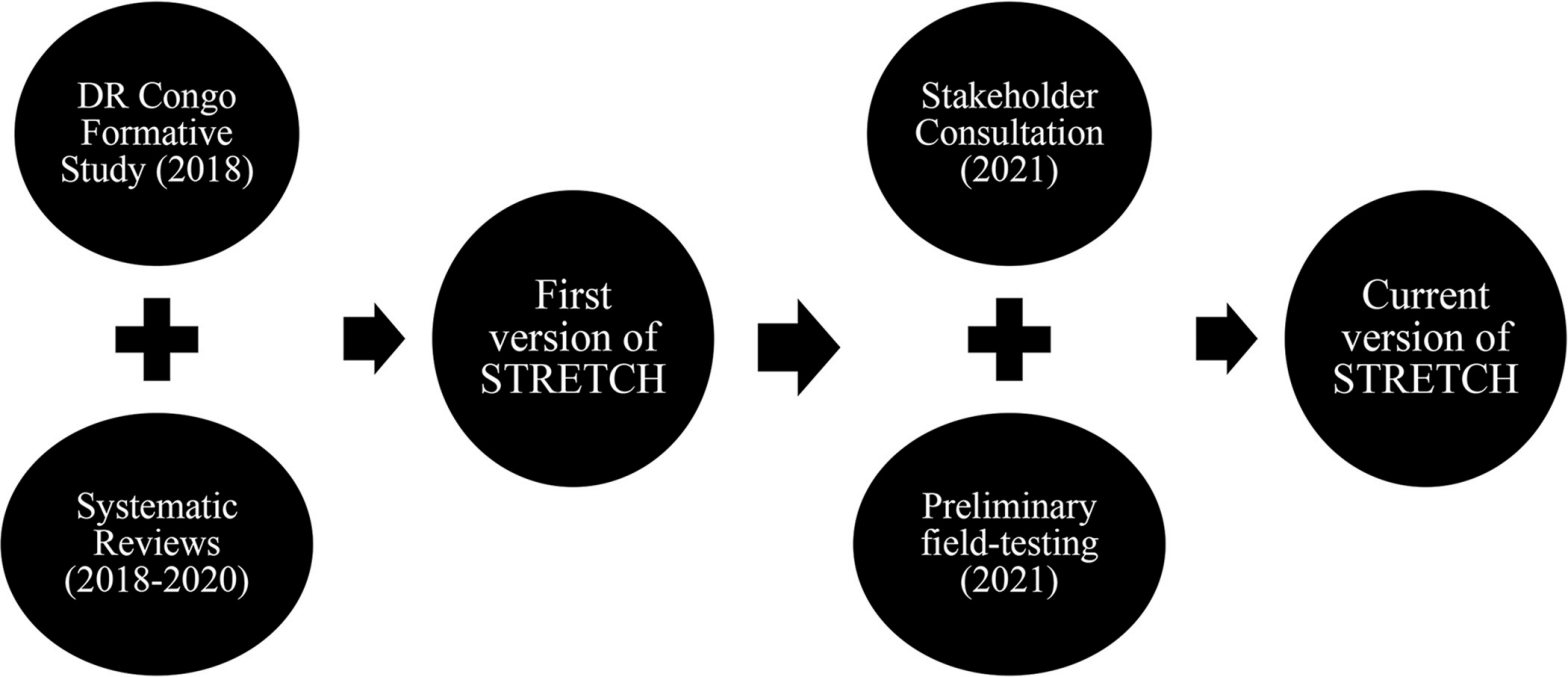
Formative research and steps to develop STRETCH.

The direct and indirect deleterious effects of stigmatisation on children and adolescents are undisputed, as well as the window of opportunity that childhood years provide concerning relationship building, self-regulation, social cognition and brain development [40]. The humanitarian organisation War Child, dedicated to improving the psychosocial well-being and resilience of children and communities affected by armed conflict, integrated stigma reduction into their Theory of Change and research agenda [41]. War Child embarked on an iterative process of stigma reduction intervention development with four main steps (see Fig 1). In this paper we aim to present the development of the intervention, drawing on previously conducted formative research and systematic review (steps 1 and 2) [42, 43] described in this introduction section, followed by stakeholder consultation and preliminary field-testing of intervention components (steps 3 and 4), reported within this paper.

As part of continuous literature scoping, a systematic review [42] to identify stigma reduction interventions across stigmas in LMIC with a focus on children and adolescents, was one of the first activities undertaken. The review confirmed that children and adolescents were underrepresented in stigma reduction interventions in Low- and Middle-Income Countries (LMIC), with 23% of the included studies targeting or impacting adolescents. This review further demonstrated that most child-focused stigma reduction research was restricted to HIV/AIDS or mental health stigma, while children and adolescents are, like adults, impacted by more distinct stigmas such as teenage motherhood [44], association with armed forces and groups [45] and albinism [46]. A third conclusion drawn from this review was that most stigma reduction interventions, for children and adults alike, address stigmatisation at one socio-ecological level, while research has indicated the importance of addressing stigma at multiple levels [27, 47]. Lastly, our review demonstrated that stigma reduction interventions applied a limited set of strategies, with similarities across stigmas, amplifying the value of cross-stigma research and interventions, as recommended [48, 49]. This review, supported by other research, led to the decision to develop a new common intervention, informed by other interventions, instead of adopting or adjusting an existing single-level and single-stigma intervention. This intervention intended to (i) have children and adolescents both as target and impact group; (ii) be applicable across stigmas, increasing its relevance as set out in recent research that advocates for moving away from stigma-siloed approaches [48, 50]; (iii) apply a combination of promising and often-used stigma reduction ingredients such as social contact [25], knowledge and awareness raising [51], popular opinion leaders [52] and empowerment of PWLE [53]; (iv) address stigma at multiple levels within the community–from targeting PWLE to strengthen their stigma resistance and coping to the community at large—to be most effective [2, 27, 48] and; (v) be implementable by non-specialists to increase its usability [54, 55]. The conclusions on cross-stigma applicability and the need for a socio-ecological response were confirmed by qualitative research carried out in DR Congo, which additionally highlighted the need for a simplified contexualisation tool [43].

The above steps led to the initial version called STRETCH (Stigma Reduction to Trigger Change for Children), inspired by the Health Stigma and Discrimination Framework (HSDF) [48] as a four-phased intervention estimated to be implemented in six to nine months. Phase 1 focused on stigma reduction among staff from the implementing organisation and the selection of a stigma to address; Phase 2 focused on understanding the context–e.g. drivers, manifestations, socio-ecological levels–of a specific stigmatised characteristic through the implementation of a specific process, called StigMapp; Phase 3 targeted (in)formal community leaders with and without the stigmatised characteristic with parallel stigma reduction strategies and then bring them together to get acquainted and engaged in regular community activities; and Phase 4 intended to be implemented by a collaboration of community leaders and the implementing organisation, to target those places in the community where stigmatisation occurs, with adapted versions of predeveloped stigma reduction strategies. This version was the starting point for the two intervention development steps presented in this paper. S1 Table provides details of each phase of this initial version in comparison to the final version of STRETCH, with the main adaptations highlighted.

## Materials and methods

This paper presents the two process steps in the development of STRETCH, a multi-component stigma reduction intervention applicable across stigmas and settings. The steps discussed in this section are (i) stakeholder consultation; and (ii) preliminary field-testing.

### Step 1: Stakeholder consultations

#### Ethical approval

Ethical approval was provided by the internal ethics approval committee of War Child, under whose authority STRETCH is being developed.

#### Research design and methods

Stakeholder consultations were organised to solicit feedback on the initial version of STRETCH in its entirety and two specific STRETCH components. Consultations were divided into four categories and conducted by the first author [KH].

#### Qualitative

Category 1 focused on ‘high-level feedback’ on the initial version. In a two-hour session, STRETCH was presented through short videos and additional explanations. Participants could ask clarifying questions. The four guiding questions for feedback were: (i) ‘what is good about STRETCH to reduce stigmatisation?’; (ii) ‘what needs to be removed due to potential harm or lack of evidence?’; (iii) ‘what are implementation concerns?’; and (iv) ‘what are suggestions for adaptation?’

In category 2 the entire intervention was presented, in three sub-sessions of three hours each. The participants were asked to react immediately during the presentation, with the same guiding questions as category 1.

Category 3 focused on a process to facilitate contextual understanding of drivers, manifestations and places of stigmatisation, to strengthen both contextualisation and relevance of the intervention. Per participant, a 2.5-hour session was conducted. The entire process was discussed: qualitative and quantitative data collection exercises, offline formats to facilitate note-taking, online data insertion and online visualisation to support the interpretation of the data.

Category 4 finally concerned a card game called Community Tales, developed as a tool for potential implementing organisations to reflect on stigmatisation and create a high-level understanding of STRETCH. The participants played Community Tales in small groups, for an estimated 1.5–2 hours. Participants were asked if and how to adapt the game.

#### Quantitative

The participants of categories 1 and 2 were asked to complete a survey after participation, to capture their perceptions concerning the appropriateness, acceptability, feasibility and scalability of the initial version of STRETCH. Three instruments with good psychometric properties in other research [56] were used to measure intervention appropriateness (IAM), acceptability (AIM) and feasibility (FIM). Potential scalability was measured with an adjusted version of the QUALIDEC measure [57] to have the same format as the other measurements. The adjustment was endorsed by the creator of QUALIDEC.

#### Study population and sampling

Inclusion criteria for categories 1 and 2 were being i) a stigma reduction practitioner or researcher and ii) from LMIC or having LMIC experience. We aimed for diverse representation across LMIC contexts and stigma backgrounds. Purposive sampling was applied: potential participants were identified through existing networks of the authors or identified through Linked-In, ResearchGate and stigma reduction articles. Associations of PWLE were specifically approached. Inclusion criteria for categories 3 and 4 were being a practitioner in humanitarian settings; no stigma reduction experience was required. To identify these participants convenience sampling was applied, using the existing networks of the first author [KH].

#### Procedures

The participants were approached and requested if they wanted to participate in a specific category. Per category, a participant information sheet was developed and shared with the request for participation. Participants were able to ask questions regarding the study, and indicate their convenience. Informed consent was collected digitally or online. Each session was recorded and transcribed. This study took place during Covid-19; all consultations were held online.

#### Data analysis

The qualitative data were analysed by the first author (KH) using thematic analysis, conducted in NVivo12. The four guiding questions provided deductive themes (good; requiring removal; triggering concerns; and adaptation suggestions) concerning the STRETCH phases and components. Simultaneously, themes cutting across these components and phases were inductively identified. Preliminary qualitative analyses were shared with the participants for feedback and validation, and one validation workshop to ask for responses on the main findings was held to provide further insights on proposed adaptations. Quantitative data were analysed descriptively in Excel.

### Step 2: Preliminary field-testing

While restricted by Covid-19, we managed to preliminary field-test two STRETCH strategies with humanitarian staff–colleagues of War Child and a partner organisation—in Uganda. Five exercises of the STRETCH strategy focusing on organisations and service providers were transferred to an online environment and tested with two groups of humanitarian staff (n = 30 in total). The participants could provide feedback through an online survey and during the session. Secondly, two online training sessions and one refresher session were held in preparation to test the card game Community Tales, as mentioned in category 4, onsite in a refugee setting in Uganda. As part of regular programming, four rounds were conducted with more than twenty community members by three facilitators. The facilitators shared their implementation experiences and the content of the discussions in writing, while a colleague from the implementing organisation overseeing this process was informally interviewed by the first author [KH].

## Results

The results will be described in three sections. Section 1 describes *which learnings* were derived from stakeholder consultations; Section 2 describes the feedback from preliminary field-testing. Section 3 details how these steps *informed the adaptation* of the initial into the final version of STRETCH.

### Section 1: Stakeholder consultation

#### STRETCH consultation

**Fig 2:**
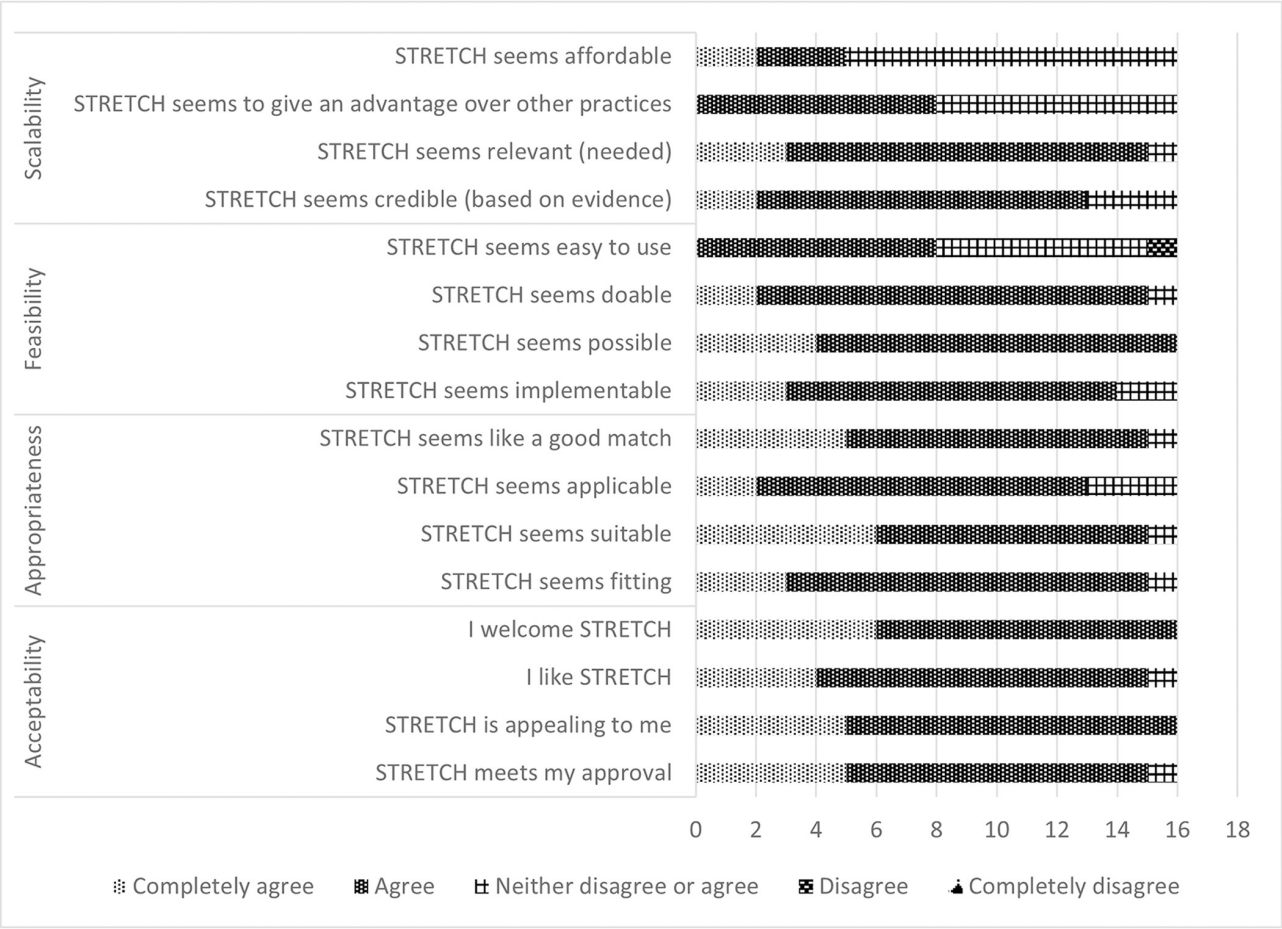
STRETCH acceptability, appropriateness, feasibility and scalability (n = 16).

Twenty-five practitioners and researchers participated in category 1 and two in category 2 consultation sessions; 63% were female and three of the participants disclosed they experienced stigmatisation themselves. The participants had experience across a wide variety of contexts and stigmas; multiple participants had experience in more than one stigma and more than one country. See Table 1 for more information. Sixteen of these participants (59%) filled in the survey to assess STRETCH acceptability, appropriateness, feasibility and scalability. In general, 97% found STRETCH (completely) acceptable, 91% found it (completely) appropriate, 83% found it (completely) feasible and 64% found it (completely) scalable. See Fig 2 for more details. This quantitative data, supported by qualitative data, highlighted that specific attention needed to be given to the scalability of STRETCH, strengthen the perception of advantage over other stigma reduction interventions, and further investigate the affordability of STRETCH implementation.

**Table 1:** Stakeholder consultation participant details (category 1 and 2 sessions).

*Stigmas represented by the participants (n = 27)*		
Researchers (51%)		
Female (66%)		
Participants disclosing having a stigmatised identity (11%)		
Health-related stigmas	Non-health related stigmas	Overarching
• Neglected Tropical Diseases, often leprosy/ Hansen’s disease (n = 6) • HIV (n = 6) • Disabilities (n = 3) • Albinism (n = 1) • Tuberculosis (n = 1) • Skin conditions (n = 1) • Mental Health (n = 6) including substance abuse	• LGTBQI (n = 3) • Gender/ sexism (n = 2) • Racism (n = 2) • Safe abortion (n = 1) • Adolescents contraception (n = 1) • Occupation (n = 1) • Sexual Rights and health (n = 1) • Sexual violence (n = 1)	• Social stigma (n = 2) • (Healthcare workers) self-stigma (n = 2) • Intersectional (n = 1)

In general, the participants seemed to appreciate the initial version of STRETCH for (i) being a comprehensive multi-level intervention; (ii) integrating evidence-based key ingredients, specifically social contact and information, including myth-busting; (iii) involving PWLE; (iv) recognising potential stigmatisation by the implementing organisation and addressing that early in the intervention and; (v) the focus on contextualisation and local relevance.

Importantly, implementation concerns, adaptation suggestions and important stigma reduction themes were defined and are described below. Additional details, including quotes and how this feedback informed the adaptation of the initial version of STRETCH to its final version, can be found in S2 Table.

Major implementation concerns were (i) the duration of the intervention or specific exercises and sessions, with participants indicating they were either too short or too long; (ii) potential stigma by association for participants due to their involvement with PWLE; (iii) the commitment of the service provider to implement the intervention as is, additional to their daily tasks; (iv) hierarchy between people ‘with stigmatised identities’ and ‘without’; (v) distinguishing between ‘stigmatiser’ and ‘stigmatised’, as the reality is not binary; (vi) that intentions to reduce stigmatisation can also be harmful; and (vii) the extended engagement of community leaders as they might not have the time.

Suggestions for adaptations were, among others, to: (i) facilitate the target group to take actions to reduce stigmatisation, by providing checklists and examples; (ii) strengthen the understandability of the intervention setup and content for potential implementing organisations; (iii) strengthen the understanding of power dynamics as key to stigmatisation; (iv) strengthen earlier and stronger involvement of families and people close to PWLE; (v) focus on practical abilities to reduce self-devaluation; (vi) set monitoring moments regarding the actions identified by the implementing organisation to reduce stigmatisation; and (vii) strengthen the flow and underlying theory of the I-STRETCH strategy as part of STRETCH, specifically targeting PWLE.

Inductive analysis led to several overarching themes. These were (i) to take into account intersectionality, as people can experience stigmatisation due to multiple identities; (ii) to strengthen the involvement and influence of stakeholders and PWLE at the earliest possible opportunity; (iii) to provide more information on facilitators’ competence and profile, such as mental health experience and dealing with resistance; (iv) to reflect on the sustainability of outcomes, and identify whether additional activities are required for sustained change; (v) the challenge of the promising strategy social contact, especially when a stigma is invisible, as it requires people to disclose; (vi) to include children and adolescents more intensively as a target group; and (vii) whether an adaptable, common intervention is feasible. An additional theme did not discuss content but research feasibility: how to measure the effectiveness of STRETCH due to its multi-level character. For further details how these themes influenced STRETCH development, please see S2 Table.

#### StigMapp stakeholder consultation

In category 3, five sessions with one participant each were held to discuss StigMapp—the data analysis process developed in response to the DR Congo formative study—to simplify the method for contextual understanding. Participants came from Colombia, Uganda and the Netherlands. While appreciated, feedback revealed that the process of StigMapp was too complicated as well. Although after the stakeholder consultation we simplified StigMapp by removing a lot of detail, also with these simplifications, implementation remained tedious. In the final version of STRETCH, StigMapp has therefore been removed and replaced by Community Tales (see below) as creating insight into pertinent stigmas remained crucial for contextualising stigma reduction efforts.

#### Community Tales stakeholder consultation

Within category 4, to test the card game Community Tales, four sessions were held online with ten participants in total, which means two sessions with three persons and two with two persons. For pragmatic reasons, one additional session was conducted with one participant to review the suggested adjustments. Participants came from Uganda, Colombia, Lebanon and Syria. Based on the feedback, Community Tales expanded, both in content and use. A card deck was added to support reflection on *the consequences of stigmatisation*, and the order of the cards was changed to create a better flow in the discussion. Additionally, next to its intended use to explain STRETCH as an intervention, two spin-offs of the game were created: Community Tales-Coping, for PWLE with a focus on coping strategies and resisting stigmatisation, and Community Tales-StandUp, for individuals to identify which actions they could take when witnessing stigmatisation. Community Tales has become an integral part of other STRETCH strategies; I-STRETCH, Inter-STRETCH for people close to PWLE and Team-STRETCH, for organisations and service providers.

### Section 2: Preliminary field-testing of STRETCH strategies

Five of the eleven exercises of Team-STRETCH were conducted in three online sessions with two groups (varying between 10 and 20 participants). Reflection concerning the exercises and their content demonstrated that these reflective exercises created insight into stigmatisation, with participants sharing examples they had witnessed in their environment. It also triggered reflection concerning their own identities and how these identities could influence attitude and behaviour towards others. Subsequently, three online training sessions were held with Ugandan humanitarian practitioners (n = 15), to implement the adjusted Community Tales with small community groups (n = 5, between 4–6 pax), as part of regular programming. Within the sessions, community members reflected among others on Covid-19, HIV/AIDS and suicidal ideation stigma, discussed its existence, the consequences, their potential role and the need to reduce stigmatisation. The insights gathered during the Community Tales sessions were translated into six radio talk-shows, which were not originally planned in STRETCH, to strengthen wider community reflection. Anecdotal reporting indicated that Community Tales and the outreach through radio seemed to trigger community demand for stigma reduction. This led to a more central position of Community Tales in STRETCH, with the card game used for multiple purposes. First, Community Tales is used for its original purpose: reflection by the implementing organisation about stigmatisation while learning about STRETCH. Second, Community Tales is now used as a stand-alone strategy, a conversation starter and reflection tool with several community groups with minimum time investment. Third, the implementing organisation can use the Community Tales scenarios as content, after learning from the community conversations and reflections held on the stigma(s) of concern, for the Community Outreach strategy, in which two-way communication with the community is intended. A Community Outreach strategy was added based on the radio talk-shows held, with the assumption that the amplified reflection and awareness will lead to various people requesting stigma reduction activities; for themselves if they experience stigmatisation, within families who include people facing stigmatisation, with service providers who realise that they need to make changes to improve the reach and quality of their work, and with community leaders who recognise they can play a role. This change led to STRETCH becoming more community-requested or community-driven than initially foreseen.

### Section 3: STRETCH adaptations

**Fig 3:**
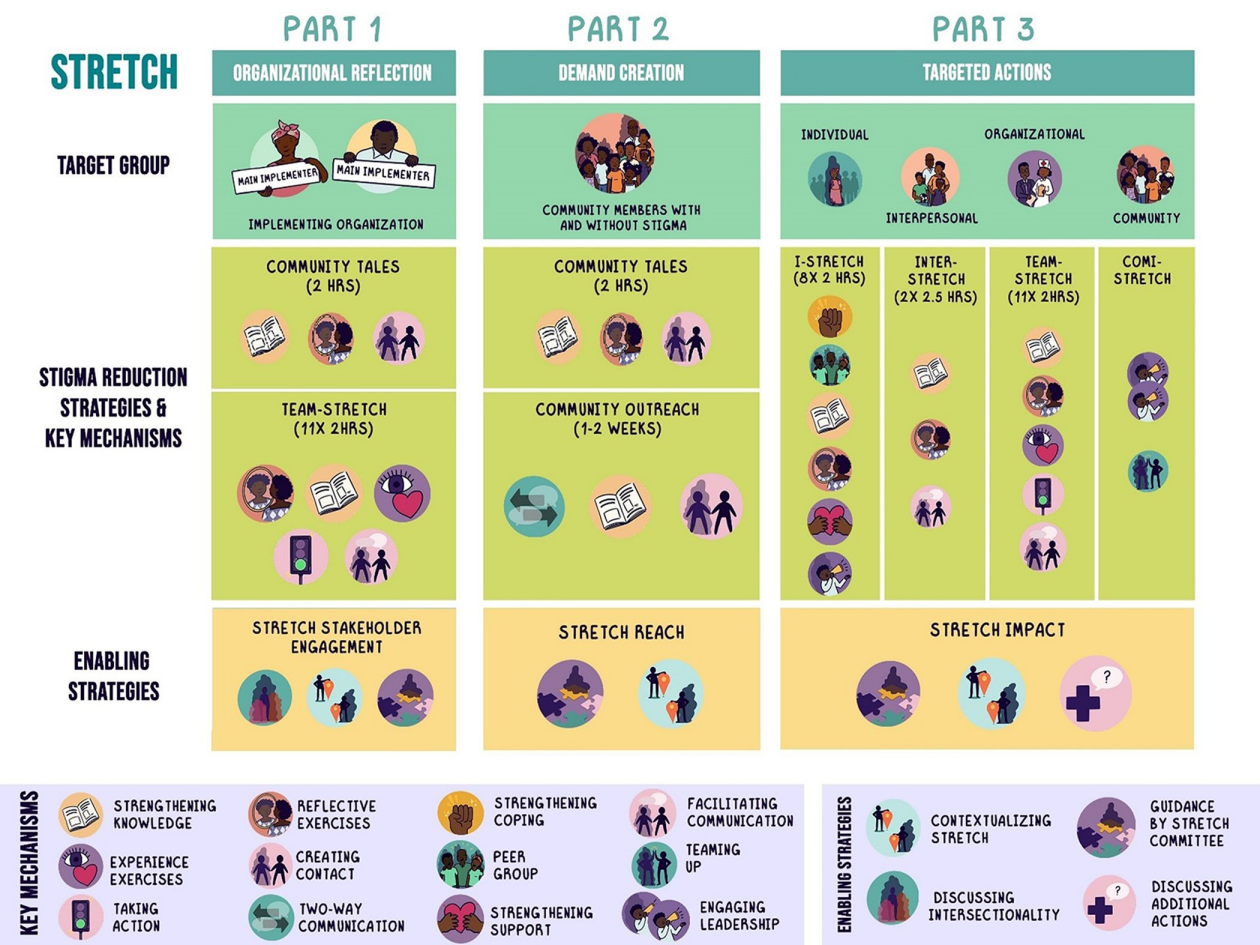
Visualisation of STRETCH final version.

While retaining core elements such as impacting children and adolescents, stigma reduction within the implementing organisation, the cross-stigma applicability, intervening at multiple socio-ecological levels and integrating various promising strategies, STRETCH has been simplified as a result of the development process. From a strict four-phased initial version, the final structure of STRETCH is more flexible to implement. The complex data collection and interpretation process StigMapp has been replaced by Community Tales, a 2–3 hr card game, and the role of community leaders has been reduced, though retained, for reasons of feasibility. PWLE and other stakeholders have been given a stronger position in guiding and contextualising the implementation. This, and community-wide communication and reflection on the stigmatised identity/ies to generate demand to reduce stigmatisation, hase made STRETCH a more community-driven intervention. Due to the repetition of some strategies (Community Tales, Team-STRETCH), preparation and contextualisation time have been reduced. Lastly, while STRETCH is developed to be implemented in its entirety, stakeholder feedback made clear that the implementation of a multi-level, multi-component, multi-month intervention will not always be feasible. STRETCH will be presented in such a way that its components can be implemented separately if the situation so requires. STRETCH is visualised in Fig 3; more details on the content of the separate STRETCH strategies can be found in Table 2.

**Table 2:** Structure and content of STRETCH strategies.

*Summary per STRETCH strategy*			
*Strategy (number*: *type of participants)*	*Estimated* *duration*	*Stigma reduction ingredients*	*Content and setup*
Community Tales (3–6: community members with and without the stigma; management of implementing organisation)	1*2hr session	• Information-based • Contact-based (imagined)	A card game guiding players to reflect on: (i) a positive outcome they want to create; (ii) the stigmatised identity/ies and name of the ‘main character’; (iii) where stigmatisation can occur; (iv) by whom stigmatisation can be conducted; (v) how stigmatisation can manifest; (vi) why stigmatisation can happen; (vii) consequences of stigmatisation; (viii) potential actions to address the source or stress of stigmatisation. Reflection moments are included. The game ends by imagining positive/pleasant contact with (someone like) the main character, leading to personal resolutions. For PWLE this short imagination exercise concerns resisting stigma.
I-STRETCH (10–12: PWLE)	8*2hr sessions, including optional disclosure session.	• Information-based • Peer group-based	Session (1): Identity; (2) & (3): Coping mechanisms (Community Tales-Coping); (4): Myth-busting; (5): Disclosure strategies (optional; based on the stigma); (6) Inspirational peer learning; (7) Strengthening the support base; (8) Positive perspective. Including monitoring/follow-up visits.
Inter-STRETCH (4–10: people close to PWLE)	2*2.5 hr sessions.	• Information-based • Contact-based (testimonial, conversation, dialogue).	Session 2: Exercise (1): Community Tales-StandUp/Coping. Discussing (dealing with) stigma (by association). Session 1: Exercise (1): experiencing exclusion; (2) myth-busting; (3) & (4) (in)direct testimonial, facilitated dialogue.
Team-STRETCH (10–16: service providers)	11 exercises, in total 3 days. Can be implemented in separate sessions.	• Information-based • Contact-based (conversation)	*Stigmatisation process*: Exercise (1): identity *; (2) prejudice and influence on attitude/ behaviour *; (3) experiencing exclusion; (4) Stigmatisation, consequences and actions (Community Tales-StandUp)*. *Stigmatisation constructs*: (5) intersectionality*; (6) privilege; (7) personal experience; (8) power dynamics. *Actions*: (9) adapting activities; (10) action plan*; and (11) contact-based dialogue.
Comi-STRETCH (10–14: community influencers and PWLE, reaching more community members)	At least 1*2hr session (to get to know each other) + daily activities.	• Popular opinion leaders-based • Contact-based (contact, conversation, connection, collaboration)	Some sessions in which community influencers with and without the stigma(s) STRETCH intends to address are; connected; starting a conversation; starting collaboration; and conducting regular, daily community activities (e.g. market visits) together.
Community Outreach (unknown: community members)	1-week development, 1–2 weeks outreach	• Information-based • Contact-based (indirect, testimonials)	Awareness raising about stigmatisation, consequences and actions through local communication channels (radio, community meetings, posters). Specific guidance to reach children/adolescents. Information from Community Tales.

*Preliminary tested

STRETCH is distinct from most interventions in its ambition to be applicable across stigmas and settings. Additionally, STRETCH stands out given its focus on children and adolescents. It is a community-level stigma reduction intervention including three consecutive parts. (1) Part one *(Organisational Reflection)* focuses on stigma reduction amongst staff of the organisation implementing the intervention, applying two strategies: the card game ‘Community Tales’ and ‘Team-STRETCH’, the strategy developed for service providers and organisations. STRETCH starts with this focus on the implementing organisation to practice stigma reduction internally before facilitating a community process, as service providers and humanitarian organisations can stigmatise as well [58]. Subsequently, the organisation reaches out to PWLE, including adolescents and youth, and other key stakeholders to form a committee to guide contextualisation and implementation. (2) Part two *(Demand creation)* aims to ensure community-wide reflection about stigmatisation, its consequences and potential actions, to generate demand for stigma reduction. Two strategies are employed, the card game ‘Community Tales’ and the communication strategy ‘Community Outreach’. In both strategies, adolescents are one of the target groups. (3) Part three *(Targeted Actions)* employs various strategies to target PWLE, including adolescents and youth (I-STRETCH), people close to PWLE (Inter-STRETCH), local service providers and organisations (Team-STRETCH) and the community at large through the involvement of community influencers (Comi-STRETCH). The implementing organisation, community implementation committee and potentially other stakeholders will thereafter discuss whether additional actions are required to sustain or strengthen stigma reduction. While it is intended to implement STRETCH in its entirety, we anticipate that contextual reality may hamper this. As such, we have developed STRETCH as a modular approach as well, where different constellations of intervention components can be chosen for a specific target group, e.g. a service provider or a school setting, if so required. This setup will inform future feasibility and effectiveness studies.

## Discussion

This paper describes the process of the development of a stigma reduction intervention. During three years, literature reviews, stakeholder feedback and preliminary field-testing led to multiple iterations until its final version. This has resulted in a multi-component stigma reduction intervention to target and impact children and adolescents, intended to be applicable across stigmas in LMIC. While some stakeholders questioned whether a common, cross-stigma intervention was feasible, recent exceptions in LMIC support that it can be: the common health-related internalised stigma reduction approach for people with schizophrenia, diabetes, HIV and leprosy in Indonesia [59] and the Human Library Methodology, where students converse with a representative of persons with lived experience, applied in Turkey [60]. Moreover, recent research has advocated for more overarching stigma reduction such as the HSDF [48] or for general strategies linked to the cross-cutting “Stigma Intervention Matrix” [49].

STRETCH consists of three parts, which in its entirety target stigma reduction at the intrapersonal, interpersonal, organisational and community levels, by using promising mechanisms such as social contact, myth-busting, popular opinion leaders, and empowerment through coping. Assessing STRETCH against a recent review of six core components for effective stigma reduction interventions [31] shows that the intervention has taken these into account. First, in response to the component culture, STRETCH follows clear theories and intervention rationale, while embedding guidance and contextualisation of processes and promising strategies [61] by PWLE and other stakeholders. Second, when looking at programme design, STRETCH integrates multiple intervention methods and mechanisms, disseminated through several channels, reaching a large section of the catchment area. Third, STRETCH targets a diversity of groups at several socio-ecological levels [27], including children and adolescents. A layered response has been recommended by multiple studies [2, 27], although a recent review stated that complex interventions are not necessarily more effective [31]. Fourth, regarding the component staffing, STRETCH will be implemented by a locally embedded organisation. This organisation is the first target of STRETCH to strengthen both the legitimacy and capacity to tackle stigmatisation, and creating facilitators showcasing behaviour change as a key ingredient to reduce stigmatisation [62]. Additionally, PWLE, including adolescents and youth, and other important local stakeholders are at the core of guiding and contextualising STRETCH, an important stigma reduction principle [52]. At the end of each part, the implementing organisation and the STRETCH committee will evaluate the implementation so far, including looking at potential harms and preparing for the next intervention part. Fifth, in terms of messaging, as it is key to have targeted messages towards target groups [63], the content will be informed by local insights gained through implementation and reflection, specifically through Community Tales. Lastly, regarding the component follow-up, which looks at the outcome assessment and duration of the intervention, we recognise that implementing STRETCH in its entirety might be intensive, specifically in the understanding that most of the, single-level, stigma reduction interventions in LMIC last at most one week [42]. However, implementation can be adapted to the local situation, influencing its duration. Additionally, in the final part of STRETCH, reflections will be held on additional actions that may be required to amplify or sustain change.

We drew six main conclusions from the development of the intervention:

First, the duration of the intervention, or the session time of the underlying strategies or exercises, was repeated feedback with two main conflicting messages: the intervention, strategies or sessions were either too long or too short. We interpreted this information from two perspectives: feasibility (both for the implementing organisation and the participant) and effectiveness. The shorter an intervention, strategy or exercise, the more feasible it in principle is to implement or participate in; this was a lesson learnt in the development study of Lusli and colleagues [64]. On the other hand, the longer and more intensive the intervention, the more effective it in principle is; this has been confirmed in various studies [53, 65–67]. Within STRETCH, we have tried to address these conflicting points through (i) the integration of short activities to be conducted with multiple groups of people, and (ii) having more intensive strategies, flexible to plan and implement, with fewer groups of people. As these strategies can be ‘re-used’ with different target groups, contextualisation time for the implementing organisation diminishes. Additionally, the setup of STRETCH was made more flexible, which should allow the implementing organisation to embed STRETCH better in their activity plans. This integration aims to strengthen affordability and is an important factor for scalability [57].

Second, a recurrent point of feedback was intersectionality. Recent research has highlighted that intersectional stigmas are a common but poorly understood reality, and that it is vital to integrate intersectionality into interventions [68]. A global systematic review of systematic reviews concerning HIV/AIDS, ill mental health and physical disability stigma demonstrated that in only 17% of the reviews the construct of intersectionality was examined [69]. A recent study produced a checklist for health interventions to strengthen intersectionality, highlighting intersectionality-guiding principles such as power, reflexivity and multi-level analysis across three intervention implementation stages [70]. We have attempted to integrate multiple of these intersectionality principles into STRETCH.

Third, the importance of meaningful involvement of PWLE in the implementation of the intervention itself has been repeatedly stated. During our literature scoping, we identified a limited number of community-led or strong PWLE-involved stigma reduction interventions [71–73]. This is confirmed by a review that stated that few interventions to reduce HIV-stigma have involved PWLE in the design or implementation of the intervention [74]. A recent systematic review on mental illness stigma reduction concluded that 40% of the included interventions had PWLE involved in intervention development or delivery [31]: the review does not detail how strong this involvement is. Another recent review demonstrated promising effects of community participation in health interventions; the authors do however emphasise that there is no ‘one size fits all’ and community participation needs to be tailored to the context [75]. Within STRETCH, we have integrated the involvement of PWLE and other stakeholders through the establishment of an official implementation guiding committee. We hypothesise that this committee will strengthen local ownership [76] and will help contextualising the intervention, encourage intermittent reflection to make adjustments, and create space to identify additional actions if required for sustained change. Additionally, the community outreach strategy is intended to encourage community members, including PWLE, but also service providers and community influencers, to request stigma reduction activities as well as use their position to influence change.

Fourth, the sustainability of STRETCH was a recurrent comment. The longer-term effect of stigma reduction interventions is a common challenge, as various reviews have emphasised. Gronholm and colleagues [77] have concluded that observed effects are often small-to-moderate in regard to knowledge and attitudes, with limited evidence on longer-term impacts. Other reviews on stigma reduction interventions discussed the poor quality of studies [34, 78], which impedes conclusions about sustainability. Applying a community-driven approach is one strategy to strengthen sustainability, although concerns have been raised about the overtaxing of people’s time [79]. In STRETCH we have therefore aimed to target many people with limited time investment while including people with higher stakes more intensely. We hypothesise that targeting multiple people such as local influencers, general community members and organisations with limited time investment will lead to requests for stigma reduction activities. We hold the assumption that if stigma reduction activities are requested, people’s motivation to participate increases and the effects will be stronger and more sustained.

Fifth, an additional point of contention was the paradoxical role of social contact. While recognising the outstanding concerns regarding its evidence [34], social contact is regarded as one of the most promising stigma reduction strategies [25, 80]. However, various stakeholders, supported by research [80, 81] emphasised the constraints of community members and leaders to disclose a stigmatised identity. Social contact has remained an important part of stigma reduction within STRETCH, with the assumption that community-wide awareness of stigmatisation and support for people experiencing stigmatisation, will create conducive circumstances for people to come forward to engage in stigma reduction activities.

Lastly, stakeholders showed, while recognising that a multi-level intervention is key to confronting the power dynamics of stigmatisation [2, 27, 50, 82–84] concern regarding measuring its effectiveness, presumably due to implementation and research complexity [27]. Besides the more flexible setup of the final version of STRETCH, the underlying strategies can also be applied stand-alone. The points of feasibility and effectiveness will inform upcoming on-site mixed-methods studies regarding STRETCH, to highlight key ingredients and assess STRETCH both in its entirety and focus on separate components or intervention constellations. These studies will also provide further insights into the feasibility of an adaptable intervention across stigmas; while this has been identified as significantly challenging [49], recent research has highlighted similarities between stigmas and a more common approach to stigma research as the way forward [48, 50, 58]. Recent stigma reduction studies, albeit it intervening at one socio-ecological level, have shown promise [59, 60].

## Strengths and limitations

One of the strengths of this paper is that it describes the iterative process of intervention development of an intervention as STRETCH, bringing together the multiple studies conducted. Strengths and limitations of the systematic review and qualitative study have been described in their respective publications. In the stakeholder consultation, stigma reduction experts, across stigma experiences and contexts, with a research or practitioner background, have provided feedback before preliminary field-testing part of the intervention. These steps provided the opportunity to already adjust potentially harmful or non-effective intervention elements, before the feasibility study phase. While we are aware that the numbers of the feasibility survey were too small to draw conclusions, we do believe it provides an indication and have used it to improve the intervention.

This paper also has its limitations. While experts with lived stigma experience have provided feedback, this has been limited so far. The planned on-site studies will ensure that PWLE, among others, can provide feedback on the intervention. Moreover, the purposive and convenience sampling of participants could have led to bias, although at this stage of development we do not feel that as particularly disturbing. Additionally, the data from the stakeholder consultations have been analysed and interpreted by the first author only. The main outcomes of the data have been discussed with a group of stigma reduction researchers for validation. Covid-19 restricted more robust testing of various STRETCH strategies onsite. However, they have been interpreted in their own right, through informal feedback, and influenced the setup of the intervention. As STRETCH is now developed as a final version, upcoming feasibility and effectiveness studies will prioritise this.

## Conclusions

This paper describes the iterative process of the development of the multi-component stigma reduction intervention STRETCH, intended to be applicable across stigmas and contexts. It outlines the current content and setup of the intervention and describes six key learnings. STRETCH responds to a gap in available interventions addressing stigma among children and adolescents in LMIC and is developed by building upon research recommendations and following the recent HSDF. This led to an intervention process, with predeveloped, adaptable strategies at multiple socio-ecological levels and embedded contextualisation steps. This paper specifically focuses on STRETCH development, though we believe its learnings are applicable across stigma reduction interventions. The intervention is now ready for piloting; future research will tell whether STRETCH is a feasible, effective and scalable stigma reduction intervention targeting and impacting children and adolescents, applicable across stigmas and in multiple contexts.

## Supporting information

S1 Table STRETCH initial version, adaptations and final version. (DOCX)

S2 Table STRETCH Stakeholder feedback and adaptations made. (DOCX)
