# Supplementary material for: Reducing stigma impacting children and adolescents in low- and middle-income countries: The development of a common multi-component stigma reduction intervention
Source: PLoS One. 2023 Oct 31;18(10):e0292064. doi: 10.1371/journal.pone.0292064 (PMC10617710; doi:10.1371/journal.pone.0292064)
Supplement: S1 Table — (DOCX) [file pone.0292064.s001.docx]

**S1 Table - STRETCH initial version, adaptations and final version**

| *Initial version of STRETCH: 6-9 months of implementation* | *Adaptations* | *Final STRETCH: 5-6 of months implementation* |
| --- | --- | --- |
| 1. Purpose: stigma reduction at implementing organisation  - Target: implementing organisation - Strategy: 2-3 day Team-STRETCH to identify and tackle stigmatising attitudes, praxis and policy - Enablers: participation in card game Community Tales to understand STRETCH’ relevance; process to identify the stigma to address; participation in a facilitation training; STRETCH preparation and training | - Facilitation training optional; prior to STRETCH - Community Tales has become a stigma reduction strategy next to a game only to learn about STRETCH as an intervention - Establishment of STRETCH guiding committee with key stakeholders; embedded involvement of people with lived experience | 1. Purpose: stigma reduction at implementing organisation  - Target: implementing organisation staff - Strategy: participation in card game Community Tales and 2-3 day Team-STRETCH to identify and tackle stigmatising attitudes, praxis and policy - Enablers: STRETCH preparation and training; outreach to people with stigma (including adolescents) and other stakeholders; and creation and the start of STRETCH guiding committee |
| 1. Purpose: create an understanding of the stigma  - Target: community members with and without stigma - Strategy: 2hr Inter-STRETCH to reflect on and experience stigmatisation - Enablers: participation in a 2.5hr data collection session; data analysis and interpretation (StigMapp approach) | - StigMapp removed due to length and complexity. - No strategy contextualisation required; done in part 1 | REMOVED |
| 1. Purpose: engaging community leaders in stigma reduction  - Target: (in)formal community leaders with and without the determined stigma; community members - Strategy: 1) I-STRETCH (2*8hrs) to strengthen coping parallel to 2) Team-STRETCH (2-3days) to tackle stigmatising attitudes and praxis; and joint 3) Comi-STRETCH to get to know each other, and do small daily activities together to showcase friendly behaviour - Enablers: Pre-discussion with community leaders; monitoring of community actions | - Same strategy (Community Tales) as in parts 1 and 2: no additional contextualisation - Community-wide strategy (Community Outreach) added: reaching more community members. Informed by Community Tales insights. - Informed and contextualised by the STRETCH guiding committee - Active role community leaders moved to part 3 | 1. Purpose: demand creation for stigma reduction  - Target: adolescents and adults with and without the identified stigma to address - Strategy: 1) 2hr Community Tales card game; and 2) 1-2 week Community Outreach, two-way communication and indirect contact on stigma - Enablers: contextualisation through STRETCH guiding committee |
| 1. Purpose: Targeted actions  - Implementation: implementing organisation together with community leaders - Target: individuals with determined stigma; organisations; community members - Strategy: 1) I-STRETCH (2*8hrs) to strengthen coping; 2) Team-STRETCH (2-3days) to tackle stigmatising attitudes and praxis; and 3) Comi-STRETCH to organise larger community events - Enablers: Contextualisation and preparation of strategies, parallel implementation, monitoring | - More community-driven; responding to local requests and need to take action for stigma reduction - No co-implementation by (in)formal community leaders due to feasibility concerns - More comprehensive socio-ecological approach: People close to people with lived experience (e.g. family, neighbours) added as a target group - Informed and contextualised by the STRETCH guiding committee - I-STRETCH strategy more focused on coping mechanisms | 1. Purpose: Targeted actions; responding to stigma reduction demands, proactive towards those places where stigmatisation has been identified.  - Target: adolescents/adults with determined stigma; people close to PWLE; service providers; community members - Strategy: 1) I-STRETCH (2*8hrs) to strengthen coping; Inter-STRETCH to facilitate conversation and support; 3) Team-STRETCH (2-3days) to tackle stigmatising attitudes and praxis; and 4) Comi-STRETCH - Enablers: contextualisation through STRETCH guiding committee; availability of More-STRETCH, to identify if additional actions are required to sustain/amplify change. |
