# Supplementary material for: Reducing stigma impacting children and adolescents in low- and middle-income countries: The development of a common multi-component stigma reduction intervention
Source: PLoS One. 2023 Oct 31;18(10):e0292064. doi: 10.1371/journal.pone.0292064 (PMC10617710; doi:10.1371/journal.pone.0292064)
Supplement: S2 Table — (DOCX) [file pone.0292064.s002.docx]

**S2 Table - STRETCH stakeholder feedback and adaptations made**

| **Theme** | **Quote** | **Example adaptations** |
| --- | --- | --- |
| *Implementation concerns* | | |
| Duration | - “My experience on the field when the session is more than a 90 minutes, people is that.. to be distracted by, by things, they always show loss of interest in the in the in the in the session.” - (interviewee) “I’m only concerned about the timelines within the different stages. This is the only thing.” (interviewer): “Do you think that they're too short or too long?” (interviewee): “too short.” | As part of the intervention manuals and within exercises, we have indicated that the duration is an indication, and that the local context should be guiding. Some sessions can also be broken up into multiple sub-sessions for better fit. |
| Potential stigma by association for participants | - “I was just wondering whether there is a risk that the community includes those changemakers, in the stigmatised groups, like we saw a little bit around HIV, if you would collaborate, you will be seen as positive and also be stigmatised” | In the finalised iteration of STRETCH we intend that community outreach on stigmatisation will trigger awareness and empathy for people concerned; and therefore will also support participation. |
| Commitment of the service provider | - “I was one of the healthcare providers, who has worked in public settings. So that's why like, I just see that sometimes these commitments they are very hard to get by. And then especially with service providers, they need a lot of boost and incentive or like some sort of, yeah, intervention, so that like, you know, like a divine intervention, or a miracle sometimes for them to like change certain things that they are already doing.” | While we already focused on the commitment of the service provider in the initial version, we have strengthened the procedure to strengthen their knowledge of STRETCH, their role, and their investment. |
| Hierarchy between people with stigmatised identities and people ‘without’ | - “well, I figured it could be maybe a problem if you include key opinion leaders. And they are, for example, a village leader, pairing them with someone who is stigmatised, who may be viewed as lower, in a way, maybe if you bring them together that could…?” | In Team-STRETCH, we added two exercises: one about power, and one about privilege, to think about your position in society and responsibilities that come with it. |
| Stigmatising experiences: don’t distinguish between stigmatiser and stigmatised | - “I feel like you could do more harm than good if you say like, here's the stigmatised people over here, and there's the stigmatised, because it just makes it too simple.” - “We all have so many different intersecting identities. Yeah, it's likely that everybody experiences some marginalisation in some way, and some access to power and privilege in another way.” | We have strengthened exercises throughout to support participants to think about their experiences and their roles; both as a person who may contribute to stigmatisation, and who experiences stigmatisation.  Inspired by this feedback we have expanded the Community Tales card game tool and made it core to the intervention, as a community conversation-amplifier on the topic of stigmatisation. |
| Good intentions can be harmful | - “It would be really good to have a strategy in place here for how to support those who find this exercise challenging” - “Like, even with your best intentions, you could be stigmatising. And it's about, are you open to? Are you open to being corrected? (…) The reason that we're ignorant about something is because of social structural systems where we didn't need to learn about it. Yeah. So if you're straight, you don't need to learn about LGBT issues, or stigma or fear, white you don't have to learn about racism (..) because (…) the system has produced knowledge that's only relevant to you.” | In Team-STRETCH specifically we have highlighted where more facilitators would be required, and mental health support pathways need to be identified prior to implementation. Furthermore, I-STRETCH and Inter-STRETCH have monitoring and follow up sessions.  This feedback further confirmed our intention to start the intervention with a stigma reduction *for the facilitating organisation*, to be able to reflect internally, make adjustments and be open. |
| Extended engagement of community leaders | - “I think that sometimes they're, there's a leader, and then they're so busy, you know, and then if we don't build up the base around them, it's not sustainable or scalable for that person” | While remaining important, the role of community leaders has been reduced. They are no longer one of the target groups within the intervention, but we hypothesise that community leaders will step forward to take action in the final part of the intervention thanks to community-wide reflection on stigmatisation in the previous parts. |
| Measure the effectiveness of STRETCH | - “On one hand, I celebrate the broad approach. On the other hand, I wonder how you're going to be able to measure?” - “I don't want to discourage I just think it's a challenge” | While in the initial version of STRETCH the stigma to address was selected in phase 2, this was taken out in the final version of STRETCH. As within an effectiveness study it is important to measure stigma at baseline (before intervention implementation), it is important that the stigma to address is already known at the beginning. Supportively, we assume that organisations which will use STRETCH are already clear about which stigma they would like to address. |

| *Adaptation suggestions* | | |
| --- | --- | --- |
| Integrate actionability for participants | - “….I mean, if people are into it, so yeah, so I'm thinking just maybe also giving a checklist or guidelines and really concrete suggestions, and getting them to reflect on what else they could do.” | Within Team-STRETCH we have adjusted an exercise (‘Reflective Action’) focusing on identifying actions to reduce organisational stigmatisation, by reflecting on a set of questions regarding common barriers for meaningful participation. |
| Strengthen the ‘understandability’ of the intervention | - “how to translate these different elements (…) into understandable, understandable concept for the community, because most of the time when you are developing strategy for us, we have done studies (…), it's easily understandable, but for the community or for community facilitators is quite challenging, because we are coming with new concepts, we are coming with a new approach, and even new vision (…). So for me, I think one part will be to (…) make it easier to be executed in the field, but also to think about how to make it understandable for the community facilitators.” | The card game Community Tales-STRETCH was drafted, with feedback from Ugandan colleagues, to facilitate understanding of the STRETCH guiding principles and key stigma reduction strategies. This card game was well received by implementers, and programmatically tried out with a few community groups in Uganda. Confirmed by this experience, further contextualisation of the card game is part and parcel of its use.  Additionally, we have invested in visualisation and packaging of STRETCH. This will be adjusted before the on-site feasibility study. |
| Focus on understanding power dynamics | - “There's a lot of power, the power dynamics between the service providers and the stigmatised individuals and the patients, that is also like another thing, because usually in such a setting in LMIC, service providers are basically all the way up, like in hierarchy. And then, yeah, the patient's like, basically, they're almost devoid of power. So I think like a source of stigma there, it is already created there, because of the power, the lack of power for patients” | Within Team-STRETCH, the strategy for service providers, groups and teams, an exercise on power (and privilege, see above) has been integrated to be able to have a discussion about the existence and role of power, and their role in this. |
| Strengthen the involvement of families earlier in the intervention | - “I would say it's also like more family interventions, really parents and these adolescents” | In the initial set-up of STRETCH, family members and people close-by were not consistently included. In the final structure of STRETCH, one of the strategies is adjusted and dedicated to this group (Inter-STRETCH). It can be implemented for the family members and people close-by participants of I-STRETCH. |
| Focus on practical abilities to address self-devaluation and empower autonomous choices | - “it makes a lot of sense working on concrete abilities, instead of just an empty narrative. I mean, they're kind of ‘black is beautiful’. But yeah, I mean, where, how? but when you know that you play soccer well, I mean, it's something so concrete and others may witness that and then give you positive feedback as well. (…) focus to empower people to really reconstruct their self worth because I see this self stigmatisation as a devaluation. If you enhance value, self value from an experiential approach, I think, I mean, yeah…” - I mean, I know, I totally respect their desire to conceal. And I've seen, I don't know, I think hundreds of people who've been concealing, and they said, Well, this is it, I conceal, and I'm happy, I can go dancing, I can go, I can fasten your, so I don't care. I conceal. - (…) So we may have like a short term and long term kind of approach to the assess these types of strategies in terms of, empowering to conceal, empowering to open up? And come out of the closet? I think those are issues that are worth discussing? Yeah. But I think the consequences to people in terms of their access, their participation in society is what really matters at the end of the day.” | An integrated element of I-STRETCH is the focus on identifying strengths and capacities; not only in yourself but also in others.  Additionally, we have made ‘disclosure risk/benefit’ an optional session within I-STRETCH. It focuses on supporting the participants to make their own decisions when and to whom they might, or may not, disclose their stigmatised status. |
| Integrate activities into ‘daily life’ |  | The current set-up of STRETCH facilitates further integration into the local context; to be implemented according to the implementer’s and participants’ agenda. We aim for the strategies to be ‘requested’ by community members thanks to having created reflection and outreach on the topic of stigmatisation; we assume that when people request something, it will also better fit their daily life.  Additionally, while we hypothesise that implementing the entirety of STRETCH has more sustained effect, the intervention is presented as such that also separate components can be implemented if the situation asks for it. |
| Integrate moments of reflection and monitoring | - “Because I think again, like if people haven't really thought about the issue of, you know, power and leadership and influence and a little thought that much before, I think you might need more, you know, more reflection and more kind of thought provoking questions to really get to. Yeah, to really get to something, you know, that's a bit more out of the ordinary than, yeah.” | The implementation of Community Tales (Coping or StandUp) intends to amplify reflection and discussion on the topic of stigmatisation. The additional strategies I-STRETCH, Inter-STRETCH, Team-STRETCH and Comi-STRETCH all intend to trigger reflection. Furthermore, for the main implementer we have built in reflective moments to revisit the action plan they created during Team-STRETCH. |
| The flow and underlying theory of I-STRETCH | - “What are the things that tie these exercises together? What is the flow?” | We have conducted additional scoping of the literature, and identified the Stress and Coping Framework (Lazarus and Folkman, 1984) as guiding framework. This led to adapting the order of sessions, and adaptation to fit with often-used coping mechanisms in stigma reduction interventions. |
| *Overarching themes* | | |
| Take intersectionality into account | - “But I think it's like, having somewhere where intersectionality is explicitly noted, then just because you experienced HIV stigma, it doesn't mean it's the same if you're a boy or girl.” - “So I think it's really important to bring that intersecting piece and say, we're going to talk about this one, maybe have like a Venn diagram. But the same HIV positive person also experiences a poverty, stigma and sexism and refugee stigma, whatever it is.” | Within the Community Tales card game we have added a possibility to reflect about intersecting stigmas, to create understanding on this issue. Furthermore, within the original Team-STRETCH strategy we already included an exercise to discuss intersectionality. |
| Strengthen involvement of and guidance by people with lived stigma experience  Strengthen stakeholders’ involvement from the beginning | - “The earlier on you're taking your community members and the community itself on board, the more it is likely that the ownership will eventually be there are so that is more than the community driven versus the agency driven balance there. That I that that's interesting to try to have right?” - “In that planning stage (…), you need to make sure you've got some education representatives, some, you know, it's that it's not just people who are interested in children born out of wedlock, you know, what I mean? (..) So the health people that are, you know, that are doing reproductive health education. And, you know, maybe people are engaging in transactional sex because they are so poor, they can't get food. And so maybe you need to think about bringing in business and industry groups to see how can we get people better jobs (…).” | The second part of STRETCH, after the implementing organisation has participated in Team-STRETCH, focuses on establishing a guiding committee of people with lived stigma experience and other influential stakeholders. This STRETCH committee has multiple purposes: to connect to people’s daily realities, ensure local relevance, monitor the situation in the community, and encourage influential stakeholders to make adjustments. |
| Facilitators’ profile and competence | - “My year-long co facilitator, for instance, always dressed up as this beautiful woman, and, people wouldn’t imagine her as being an HIV positive person. And where she really came from a CD4 count very low, very sick, whatever, to this beautiful, individual woman. That’s what’s important. And so that’s what challenged them also to say, if she can do it, I can do it. So it’s that role modelling, that’s also a very important part in whoever you have as facilitators.” - “You need to have like a social worker, or it can't just be like people who literally have no idea, you know, what I mean?” - “So are they going to be equipped in terms of how do you react so that the messaging is also the same in terms of how they react to any backlash that may come?” | One of the earliest activities within STRETCH is Team-STRETCH for the implementing organisation. This is intended to strengthen a reflective mindset about own and organisational attitude and behaviour, to be a more inclusive and observant implementer. Additionally, in the identification of staff for Team-STRETCH participation we make a reference to experience with stigmatisation, to strengthen the connection to the target group. Moreover, within the regular reflective sessions that the STRETCH committee and the implementing organisation have on STRETCH implementation, some exercises are built-in to reflect on dealing with resistance. |
| Ensure sustainability through different actions and processes | - “I just want to add something coming in my mind like building the strong networks of the lived experience. (…) If we build a strong networks of people with the lived experience, that they have a unified voice, isn't it?” - “Maybe you can influence advocacy organisations that are then going to go advocate for that and make that outcome.” | The adjustment of STRETCH, from a more rigid phased approach to an approach where stigma reduction activities are requested by community members, is one of the adaptations made to strengthen sustained change.  Furthermore, STRETCH now includes an activity in which the STRETCH committee and the implementing organisation discuss whether more activities then what STRETCH offers are required to strengthen sustainability. |
| Challenge for community influencers experiencing stigma to disclose and step up | - “I think it (hidden stigma) is an important issue, like how do you actually combine this kind of, you know, wanting to have the social contact element? And but then yeah, you know, tying that in with, you know, hard to reach stigma, actually, which is, I guess it's just a consideration for your project. And it might be a consideration when you think about what stigmas to address is kind of like thinking about the feasibility. But then obviously, yeah, you don't want to shy away from trying to like address statements that are really hard to reach at the same time either” - “I don't think we can find anyone in the community who would be willing to come forward and say that had a particular mental health problem” | The encouragement to use social contact as a strategy and the difficulty for community members, and leaders, to step up and disclose they have a certain stigmatised identity, is paradoxical and challenging. We did not make any adjustments to STRETCH to tackle this challenge, and will integrate this as a specific point of interest in the upcoming on-site feasibility study. However, we hypothesise that the adjusted set-up of STRETCH will cause some people to stand up, encouraging others to follow their lead. |
| Stronger involvement of adolescents as target group | - “we also need to make it very practical, and also, really people on the ground and also, if your project is about adolescence, really that's a target group, though. What about them? So what will continue after three years they will be adults? Yeah, you start at age of 15. So it's to think in that line when they become adults, so how do we, what do we expect or what is in our ideal world? Yeah, they are actually be those change agents, I would say because earlier you start easy becomes so yeah.” - “It could be considered surprising that the intervention mainly involves adults as participants and facilitators, when the intervention focuses on triggering stigma reduction among children. (…) Could there be more direct links to change attitudes among children?” | While adolescents had been part and parcel of the initial version of STRETCH, multiple times it was mentioned that it was insufficient. This was also feedback from one content reviewer after reviewing the adjusted STRETCH version. In the final set-up of STRETCH, adolescents are specifically targeted during Community Tales and the community outreach (part 3). We intend for I-STRETCH (part 3) to be also suitable for groups of adolescents experiencing stigmatisation. Additionally, the STRETCH committee will include adolescents and youth as well. |
| Feasibility of a common approach | - “I'm definitely a big believer in the, like, common at its core, you know, you know, thinking around stigma. So I actually don't see major pitfalls. Like, you know, a lot of people will probably, I mean, some people might say, like, Well, you know, this specific stigma is going to be different, because that's one issue, like, with both researchers and advocates, they want to think that their thing is their topic is special.” - “I'm just thinking, I don't know, but I'm thinking are all stigmas the same, are all interventions against specific types of stigma the same, so mental health stigma, and poverty stigma, other health stigma?” - “what does stigma mean in different cultures? And, and and yes, so how is that somehow translated in this setup” | Some stakeholders questioned whether a common or adaptable approach, an approach to be applicable across stigmas, would be feasible. Responding to reality, we have ensured that STRETCH is developed as a modular approach: one strategy can be implemented; a targeted set of strategies can be implemented; and STRETCH can be implemented in its entirety. Depending the time and resources available, the target group etc.  Additionally, in the adjusted setup of STRETCH we open up the possibility to tackle multiple stigmas at the same time. If people with different stigmas participate in I-STRETCH, the implementer can choose to do specific sessions – e.g. on condition management and myth busting – separately, or together so that participants learn across stigmas.  Moreover, this question is a specific point of interest in the upcoming on-site feasibility study. |
